# Microbial community structure in the firn associated with a ∼1000 km transect of the Eastern Antarctic Ice Sheet

**DOI:** 10.1093/femsec/fiag095

**Published:** 2026-08-25

**Authors:** James E Lawrence, Isis Cohen Nicholson, Rebecca McCerery, Laure Newby, Rosalia Calvo-Ryan, Andrew Nelson, Joseph A Graly, Elisabeth Isaksson, Andrew Hodson, David A Pearce

**Affiliations:** School of Geography and Natural Sciences, Faculty of Science and Natural Science, Northumbria University, Newcastle-upon-Tyne NE1 8ST, United Kingdom; School of Geography and Natural Sciences, Faculty of Science and Natural Science, Northumbria University, Newcastle-upon-Tyne NE1 8ST, United Kingdom; School of Geography and Natural Sciences, Faculty of Science and Natural Science, Northumbria University, Newcastle-upon-Tyne NE1 8ST, United Kingdom; Faculty of Sciences, Université libre de Bruxelles, Av. Franklin Roosevelt 50, 1050 Bruxelles, Belgium; School of Geography and Natural Sciences, Faculty of Science and Natural Science, Northumbria University, Newcastle-upon-Tyne NE1 8ST, United Kingdom; School of Geography and Natural Sciences, Faculty of Science and Natural Science, Northumbria University, Newcastle-upon-Tyne NE1 8ST, United Kingdom; School of Geography and Natural Sciences, Faculty of Science and Natural Science, Northumbria University, Newcastle-upon-Tyne NE1 8ST, United Kingdom; Department of Polar Climate, Norsk Polarinstitutt, Framsenteret, Postboks 6606 Langnes, 9296 Tromsø, Norway; Department of Arctic Geology, UNIS- Avdeling for arktisk geologi, Postboks 156, 9170, Longyearbyen, Svalbard, Norway; School of Geography and Natural Sciences, Faculty of Science and Natural Science, Northumbria University, Newcastle-upon-Tyne NE1 8ST, United Kingdom; British Antarctic Survey, High Cross, Madingley Road, Cambridge CB3 0ET, United Kingdom

**Keywords:** antarctica, cryosphere, biogeography, core, ice, distribution

## Abstract

The surface snow and shallow ice of the Antarctic have traditionally been considered biologically inactive environments with low biomass. However, recent studies have begun to describe the complexity of microbial populations present, their biogeographic distribution and local spatial variation. This study characterized the microbial communities of Eastern Antarctic high altitude plateau firn cores (up to 27 m in depth), collected along a 989 km inland transect culminating near the Southern Pole of Inaccessibility. These cores were collected in the 4^th^ International Polar Year (IPY), during a Norwegian–U.S. scientific traverse of East Antarctica. Microbial community composition varied both between different cores and within individual cores, indicating spatial and vertical heterogeneity. Patterns of variation were not consistent across cores or depths, and regional differences were also apparent at the phylum level. Differences in measured ion concentrations, particularly phosphate, also correlated with microbial diversity. Overall, firn across the high-altitude inland plateau of the Eastern Antarctic hosts a spatially structured and depth-dependent microbiome likely influenced by depositional variability and local geochemical conditions. This study highlights the complexity of microbial distribution within firn profiles, and emphasizes the importance of understanding the local microbial diversity of surface snow and shallow ice when investigating subsurface cryosphere environments.

## Teaser

An investigation into the biogeographic distribution of microorganisms across an inland, high-altitude transect of Eastern Antarctica suggests a spatially structured and depth dependent microbial profile across coring sites and with depth into the firn.

## Introduction

Antarctica has traditionally been considered a low biomass, relatively homogeneous environment with low levels of microbial diversity and activity (Malard et al. 2019, Nowak et al. 2024). Yet, conservative estimates suggest that there may be 4 × 10^24^ microorganisms present within the Antarctic Ice Sheet (Priscu et al. 2008, Cavicchioli 2015, Vincent 2000). However, despite the increasing resolution of sequence databases over time, many of these micro-organisms remain unidentified (Knowlton et al. 2013, Kim et al. 2025). Environmental factors are known to contribute to the structuring of microbial communities in Antarctic surface snow and shallow ice, which may ultimately alter the microbiome of subsurface glacial ice (Maccario et al. 2019, Hodson et al. 2021). Spatial coverage across the Antarctic remains uneven, with many surface snow and shallow ice environments remaining under-studied, relative to the continent’s geographic extent and environmental diversity (Chong et al. 2013).

Previous investigations have demonstrated diverse microbial communities from ice cores of up to 4 m in depth, close to the periphery of the Eastern Antarctic Ice Sheet (EAIS) plateau and also from cryoconite holes in the Ellsworth mountains (Segawa et al. 2010, Hodson et al. 2013), as well as other specific regions of the Eastern Antarctic (Malard et al. 2019, Parro et al. 2025, Stoppiello et al. 2023). For example, the regions of Dome Fuji and Dome C have been the focus of previous studies of microbial population structuring (Nowak et al. 2024, Parro et al. 2025, Stoppiello et al. 2023), with both geographic areas influenced by differing large-scale atmospheric circulation patterns (Parrenin et al. 2016). These studies have demonstrated spatial variation and highlighted potential associations between microbial distributions and local environmental factors. To date, there has been only one transect across the EAIS plateau, sampling areas influenced by the marine environment closer to the coast, but no investigations deeper than 4 m (Parro et al. 2025). The high-altitude inland plateau of the EAIS experiences distinct atmospheric and depositional conditions, created by large-scale katabatic wind systems, transporting surface snow towards the coast in a predominantly downslope direction (Wang et al. 2023, Cable et al. 2026).

Surface snow communities are known to vary geographically (Malard et al. 2019), while seasonal dynamics influence surface snow microbial community composition (Stoppiello et al. 2023), and a transect across the EAIS plateau inland from the coast revealed both geographically structured microbial variation and a shared regional core microbiome, with evidence for proximity to the Southern Ocean influencing microbial community composition (Parro et al. 2025). Across the EAIS, cryoconite holes act as localized hotspots for microbial biodiversity (Nowak et al. 2024). Associations between variability in the local environmental ion concentration and variation in the microbial communities present has also been reported (Nowak et al. 2024, Parro et al. 2025, Nazaries et al. 2013). Across these environments, several phyla have been reported as ‘dominant’ taxa, including Proteobacteria, Firmicutes, Actinobacteria, Bacteroidetes, and Cyanobacteria.

To date, investigations of the EAIS have focused on the surface habitats, between the top layer of snow and ∼4 m in depth (Malard et al. 2019, Nowak et al. 2024, Parro et al. 2025, Stoppiello et al. 2023). Little is known about the microbial communities present at greater and intermediate depths in the firn, where cells arriving at a location could be subjected to selection and sorting during firn formation. Firn, an intermediate stage of snow compaction and glacial ice formation, covers most of the Antarctic Ice Sheet (Van den Broeke 2008), with microorganisms deposited from snowfall and wind-blown material becoming progressively incorporated into the firn through ongoing accumulation and densification (Harding et al. 2011). The thickness of firn across the EAIS plateau often exceeds 100 m in depth before transitioning to glacial ice (Van den Broeke 2008). Microorganisms within firn may be preserved over extended periods of time, whilst becoming further isolated from direct atmospheric influences (Zhong et al. 2018). These microbial populations within the firn column may associate with historical atmospheric deposition, specific meteorological events and temporal variation in environmental conditions (Zhong et al. 2018). The retrieval of firn cores at depths of up to 27 m from the high-altitude inland plateau of the EAIS presents a rare opportunity to examine a previously underrepresented cryosphere environment.

The firn cores analyzed in this study were collected during the joint Norwegian and United States IPY transverse across the Eastern Antarctic Plateau. Anschütz et al. (2011) has characterized the accumulation rates, stratigraphy, and geochemical properties of samples from this transverse, providing the depositional framework for microbial observations. As a result, microbial assemblages from specific depths within the firn cores may be compared to volcanic horizons, for example allowing for the influence of extreme environmental events on population structures to be determined.

Overall, this study aimed to investigate the biogeographic distribution and depth-associated variation of microbial communities within inland high-altitude firn of the EAIS. Specifically, it aimed to a) compare the microbial community structure across the high-altitude interior of the EAIS, b) assess changes in microbial community structure with depth in the firn, c) quantify local ion concentrations and evaluate their association with observed microbial community compositions, and d) define and compare the dominant microbial taxa across sampled cores with reported microbiomes from other shallow ice studies.

## Materials and methods

### Sampling protocol

**Figure 1:**
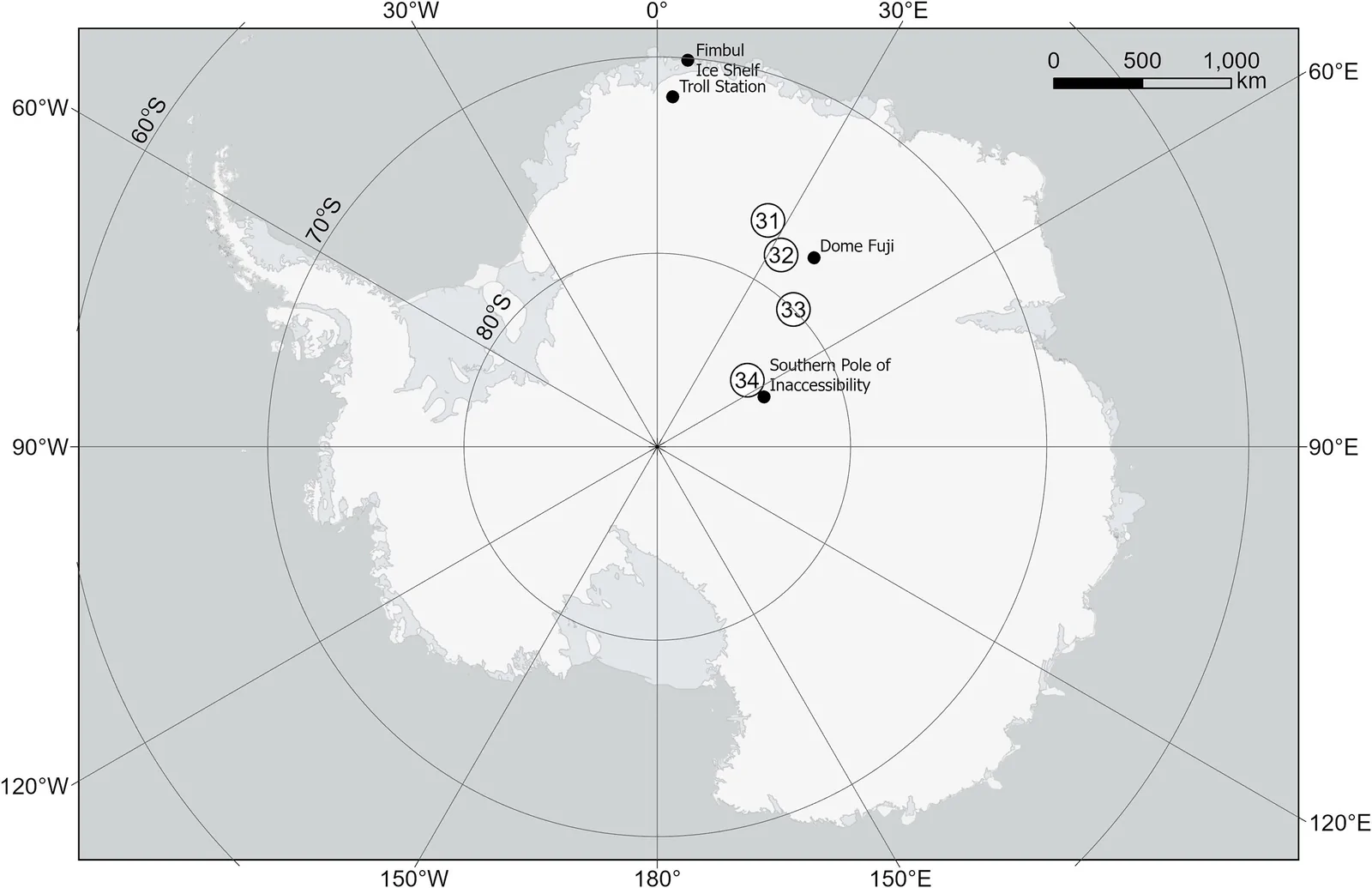
Location of sample cores on the EAIS. Dome Fuji and the Southern Pole of Inaccessibility are also shown. Basemap sourced from the Environmental Systems Research Institute (ESRI) basemap imagery in ArcGIS Pro.

The firn cores used in this study were extracted during the 2007/2008 field season of the joint Norwegian and United States IPY traverse (Winther and Albert 2019). The four cores, labelled 31, 32, 33, and 34, were high elevation (>3450 m) sites on the polar plateau (Figure 1 and Table 1). The final core length varied between 19 m and 27 m. Tyvek suits were used on-site during the drilling process. The cores were packed in plastic core tubes and sealed. Cores were kept frozen at all times between collection and analysis, stored at the British Antarctic Survey core storage facility. As no liquid was involved in the collection of the cores, the drill barrel was not extensively cleaned between samplings. Following collection, samples were handled with consideration for potential laboratory-derived contamination, with decontamination procedures applied during downstream analysis. Relationships between cores were also examined during the subsequent analysis to assess patterns of similarities between cores. The dating of these cores and accumulation rates are given in Anschütz et al. (2011). Each core was partitioned at metre intervals for analysis in the microbiology laboratory at the British Antarctic Survey before being thawed slowly at ambient temperature in a class II microbiological safety cabinet. Prior to thawing the external layer of each section was removed separately with a sterile knife. Melted central core sections were filtered through a sterile 47 mm, 0.2 µm filter using a sterile 250 ml Sartorius filter manifold attached to a vacuum pump.

**Table 1.** The coordinates of coring sites demonstrated in Figure 1, with annual accumulation (A) (estimated through Anschütz et al. 2011).

Core name	Latitude	Longitude (E)	Elevation (m)	A (kg/m^2^/a)
Core 31	−77.0	26.1	3589	Ca. 40
Core 32	−78.2	32.9	3595	Ca. 40
Core 33	−80.0	44.8	3672	Ca. 40
Core 34	−84.2	53.5	3463	Ca. 40

### DNA extraction and sequencing

DNA was extracted from thawed ice cores and blank controls using DNeasy PowerWater kits, ID 14900–100NF, and Sterivex Filter Units, Cat# SVGPL10RC, (Qiagen, Germany) according to the manufacturer’s instructions. The volume of sample depended on firn density which changed with depth, so extractions ranged from 1000 - 2650 ml in Core 31, 1000 ml - 2550 ml in Core 32, 1600 ml - 3000 ml in Core 33, and 1400 ml - 3000 ml in Core 34. Extracts were stored at −20°C before sequencing. Amplicon sequencing with Illumina MiSeq was conducted by the NU-OMICS DNA Sequencing Facility at Northumbria University according to standard protocols, returning paired-end 250-bp reads (Nelson and Stewart 2020).

Amplicon sequencing of the 16S rRNA V4 region was based on the Earth Microbiome Project Protocol (Caporaso et al. 2012) with modified primers (Apprill et al. 2015, Parada et al. 2016). Degeneracy was added to both the forward and reverse primers to remove known biases against Crenarachaeota/Thaumarchaeota (515F, also called 515F-Y (Parada et al. 2016)) and the marine and freshwater Alphaproteobacterial clade SAR11 (806R (Apprill et al. 2015)). Briefly, PCR was carried out using 1x Platinum Hot Start PCR Master Mix, 0.2 µM of each primer and 5 µl of template DNA in a total volume of 25 µl under the following conditions: 94°C 3 min, 35 cycles 94°C 45 s, 50°C 60 s, 72°C 90 s with a final extension 72°C 10 min. One positive and one negative control sample were included in each 96 well plate and carried through to sequencing. PCR products were normalized using Quant-iT™ PicoGreen™ dsDNA Assay Kit (Invitrogen) as described in the manufacturer’s instructions and pooled per 96 well plate. Each pool was cleaned using 1 : 1 Ampure XP: Pooled amplicon, quantified using fragment size determined by BioAnalyzer (Agilent Technologies) and concentration by Qubit™ 1X dsDNA High Sensitivity assay kit (Invitrogen). Pools were combined in equimolar amounts to create a single 4 nM library then denatured using 0.2 N NaOH for 5 min and diluted to a final concentration of 5 pM, supplemented with 15% PhiX and loaded onto a MiSeq V2 500 cycle cartridge.

Processing and analysis of sequencing data was performed in RStudio (R version 4.4.0). Quality filtering and trimming, clustering, removal of chimeras, and construction of amplicon sequence variant (ASV) table were performed using the Divisive Amplicon Denoising Algorithm 2 (DADA2) pipeline (Callahan et al. 2016). Forward reads were trimmed to 200-bp, and reverse reads to 100-bp based on an analysis of the quality scores. Taxonomy was assigned using the SILVA v138 database (Quast et al. 2012, Yilmaz et al. 2014) and the naïve Bayesian classifier method (Wang et al. 2007). The Decontam package (Davis et al. 2018) was then employed to minimize the influence of potential contaminants from the dataset that may be associated with laboratory reagents, extraction kits or other sources of laboratory contamination. Control samples were incorporated into the analysis, and taxa identified as potential contaminants using a threshold of 0.1 were removed prior to downstream microbial community analysis.

### Statistical analysis

Taxonomy, metadata and count data were combined into a phyloseq (version 1.48.0) object (McMurdie and Holmes 2013). ASVs assigned to chloroplast at the order level or mitochondria at the family level were removed. To simplify the data for downstream analyses, ASVs were aggregated at each taxonomic level (from phylum to genus). Relative abundances at each taxonomic level were calculated to compare microbial profiles of each core and depth sampled. The presence of taxa classified at the phylum and order ranks were visualized using ggplot2 (version 3.5.1 (Wickham 2016)) by calculating the percentage of sites where each taxon was identified.

### Minimum read counts and read difference analysis

To test for differences that might arise from differences in the number of reads between cores, a Kruskal–Wallis rank sum test was carried out. Alpha and beta diversity metrices are sensitive to differences in sequencing depth which can lead to misleading conclusions driven by technical variation rather than true biological differences (Hong et al. 2022, Schloss 2024). To test whether sequencing depth influenced the results of alpha and beta diversity measurements, minimum read count filters were applied to samples at different levels (0, 1000, 2000, 5000, and 7500 reads). For each minimum read count filter, diversity measures were then analyzed using the raw data (no further processing or data normalization) and data rarefied to an even depth based on the lowest read count. Rarefaction has been shown to be the most robust way to account for uneven sequencing depth in amplicon data (Hong et al. 2022, Schloss 2024). Using the different minimum read count filters and processing methods allowed the robustness of biological conclusions to be assessed. For the retention of reads without the sacrifice of data quality, minimum read count filters of 1000, and 2000 reads were used. Therefore, to maximize sample retention, a filter of 1000 reads was used for most statistical analyses in this study.

### NMDS

Non-metric multidimensional scaling (NMDS) was used to explore community composition of ASVs using the Bray–Curtis dissimilarity index, with up to 100 random starts. Separate PERMANOVA tests were conducted using the vegan package (version 2.6.10 (Oksanen et al. 2001)) with 9999 permutations to test the effects of site and depth. To avoid pseudo-replication due to multiple depths sampled from the same core, permutations were constrained within site for the depth PERMANOVA tests. The interaction between site and depth was also assessed. Pairwise PERMANOVA tests with Benjamin-Hochberg adjusted *p*-values for multiple comparisons (Benjamini and Hochberg 1995) identified which sites were significantly different to each other. Prior to PERMANOVA, beta-dispersal tests were used to evaluate homogeneity of multivariate dispersion among cores. If the beta-dispersal test was significant, an analysis of similarities (ANOSIM) test was also performed as this does not assume equal group variances. Separate NMDS plots and PERMANOVA tests were carried out for each minimum read depth and for raw and rarefied data. In addition, NMDS ordination and environmental fitting were performed on samples with associated chemical data. PERMANOVA tests were also carried out to assess the proportion of microbial variation correlated with identified local geochemistry.

### Association with volcanic horizons

For each core, volcanic horizons were identified using Anschütz et al. 2011. Microbial orders showing anomalous abundance at these horizons were identified by calculating the first (Q1) and third (Q3) quartiles and interquartile range (IQR) for the top 20 most abundant orders. Outliers were defined as Q1–1.5 x IQR and Q3 + 1.5 x IQR following Tukey (1977) (Tukey 1977). Any sample outside this range at a volcanic horizon was flagged as an outlier. To test whether microbial communities present in volcanic and non-volcanic horizons differed in composition a PERMANOVA test was carried out using the rarefied data (minimum read count 1000) using Bray–Curtis dissimilarity with 9999 permutations.

### Diversity indices

To assess alpha diversity between cores, pairwise comparisons of ASV richness with Shannon’s and Simpson’s indices were carried out using Wilcoxon rank sum tests (with continuity correction for richness data) for each minimum read count filter and normalization method (raw and rarefied data). The *p*-values were adjusted for multiple comparisons using the Holm method (Chen et al. 2017).

### Ion chromatography

Concentrations of ions were analyzed on a Dionex high-pressure ion chromatography (HPIC) system using 5 ml of sample and analyzed in triplicate. An eluent of KOH 10 mM was used with a pump flow rate of 0.380 ml/min at 30°C. Samples were run in triplicate with an injection volume of 2.5 µl. Standard solutions of acetate (CH_3_COO^–^), bromide (Br^−^), chloride (Cl^−^), fluoride (F^−^), formate (HCOO^−^), nitrite (NO_2_^−^), nitrate (NO_3_^−^), sulphate (SO_2_^4−^), and phosphate (PO_4_^3−^) were prepared using 18.2 MΩ/cm Milli-Q ultrapure water to obtain standard curves.

Chemical differences were assessed using Kruskal–Wallis tests in RStudio (R version 4.4.0). If Kruskal–Wallis tests resulted in significant differences observed, pairwise Wilcoxon rank sum tests with continuity correction were carried out, and *p*-values were adjusted for multiple comparisons using the Holm method. Spearman’s rank correlation was used to assess the association between depth as the concentration of individual ions within each core.

### Identification of dominant taxa

The dominant groups were defined as a ‘core microbiome’ (term not used here for clarity) i.e. taxa present in ≥80% of samples with a minimum relative abundance threshold of ≥1%. This was chosen as threshold-based definitions consistently capture shared taxa and minimize the inclusion of spurious low-abundance features (Neu et al. 2021). This follows the common practice of using high prevalence thresholds in microbial ecology (Risely 2020), accounting for the low relative biomass of this environment. Phyla was selected as this allows for a direct comparison to similar studies, and order is also shown for consistency.

## Results

### Number of reads between cores

**Figure 2:**
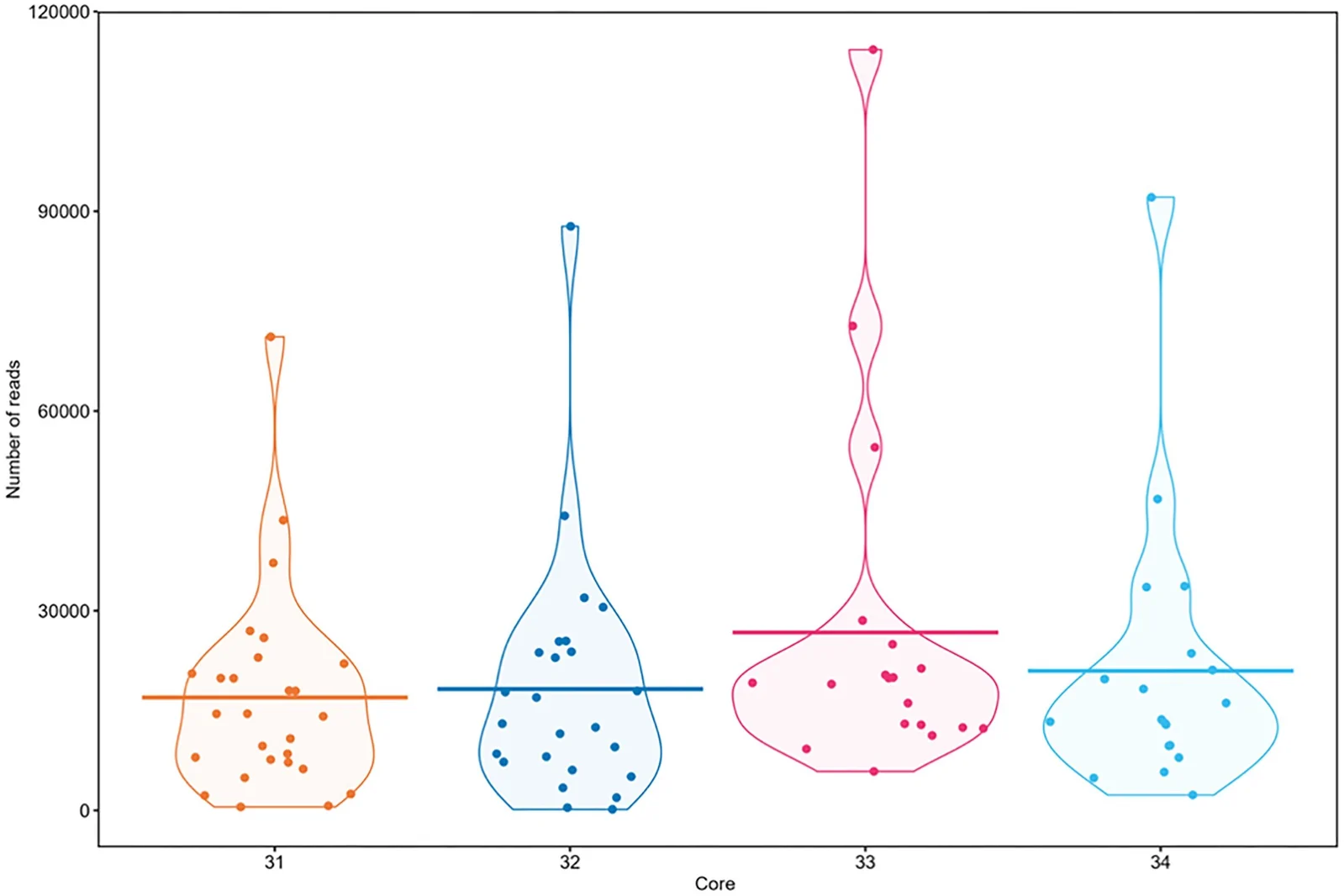
Comparison of mean and total read numbers identified across all sample cores showing no significant difference (*P* = 0.43). Any observed difference in the microbial community between cores is not due to differences in the number of reads.

The average DNA yield across all 90 samples was 0.74 pg/ml and the number of reads obtained post-sequencing and processing ranged from 157 to 114 277. However, between 10 000 and 40 000 reads were identified for most samples (Figure 2) and there were no significant differences in the number of reads between cores (Kruskal-Wallis: Χ^2^ = 2.7608, df. = 3, *P* = 0.43). After samples were filtered based on a minimum read count, 86 samples remained for the 1000 reads filter, 85 samples remained for the 2000 reads filter, 79 samples remained for the 5000 reads filter and 72 samples remained for the 7500 reads filter. At each of the minimum read count filters there was no significant difference in the raw or rarefied number of observed ASVs between cores.

### Community composition

**Figure 3:**
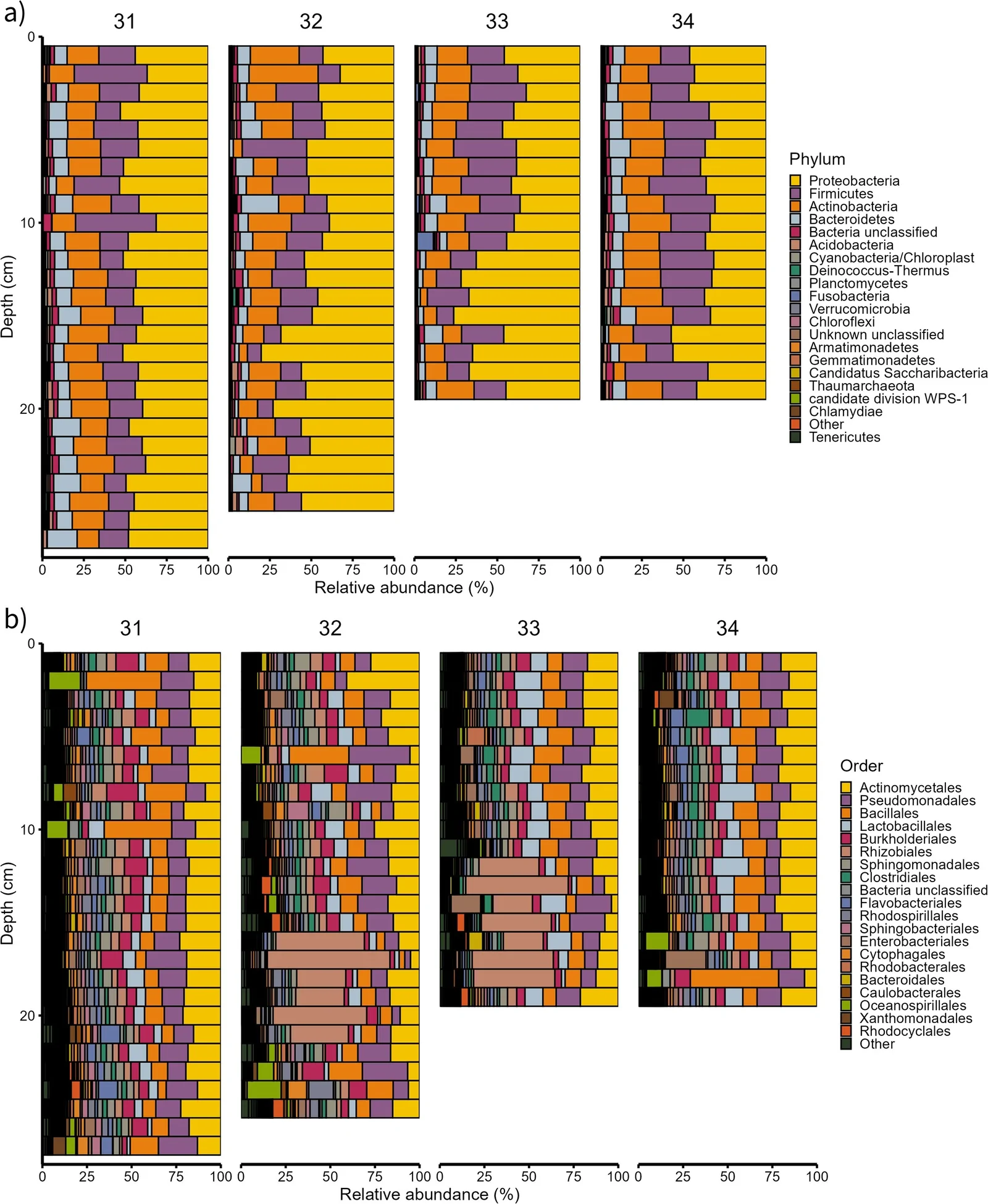
(a) Relative abundance and identification of the top 20 most abundant phyla within each core, visualized with depth into the firn. (b) Relative abundance of the top 20 most abundant orders identified within each core, visualized with depth into the firn.

**Figure 4:**
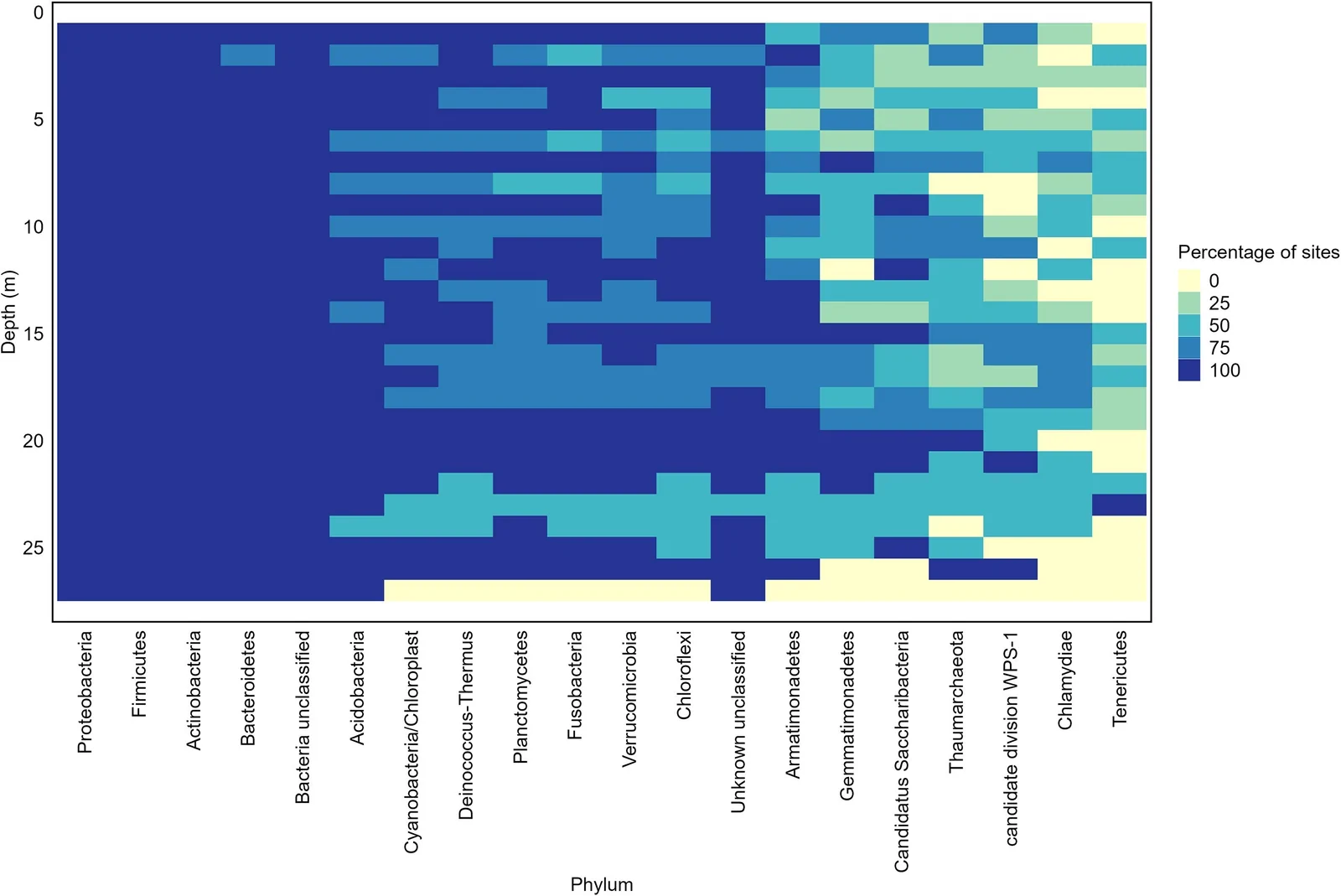
Heat map showing the percentage of samples containing identified phyla, against the core depth, arranged in order of overall identified abundance. Each datapoint represents a depth, with the colour related to the percentage of sites that contain that phylum. At shallower depths, the proportion of phyla that were identified across all cores was greater than at increased depths. The key members of the dominant microbiome (Proteobacteria, Firmicutes, Actinobacteria, and Bacteroidetes and Actinomycetales) were present across all samples, except for Bacteroidetes, which was not identified in one core at 2 m in depth.

In total, 1329 ASVs were assigned to 187 orders within 39 phyla. Figure 3 shows the 20 most abundant phyla and orders identified, and a full list of identified phyla and orders is available within the supplementary information (SIF1 and SIF2). Figure 4 shows the coverage of the twenty most abundant phyla across samples, by depth.

### Between core comparison

**Figure 5:**
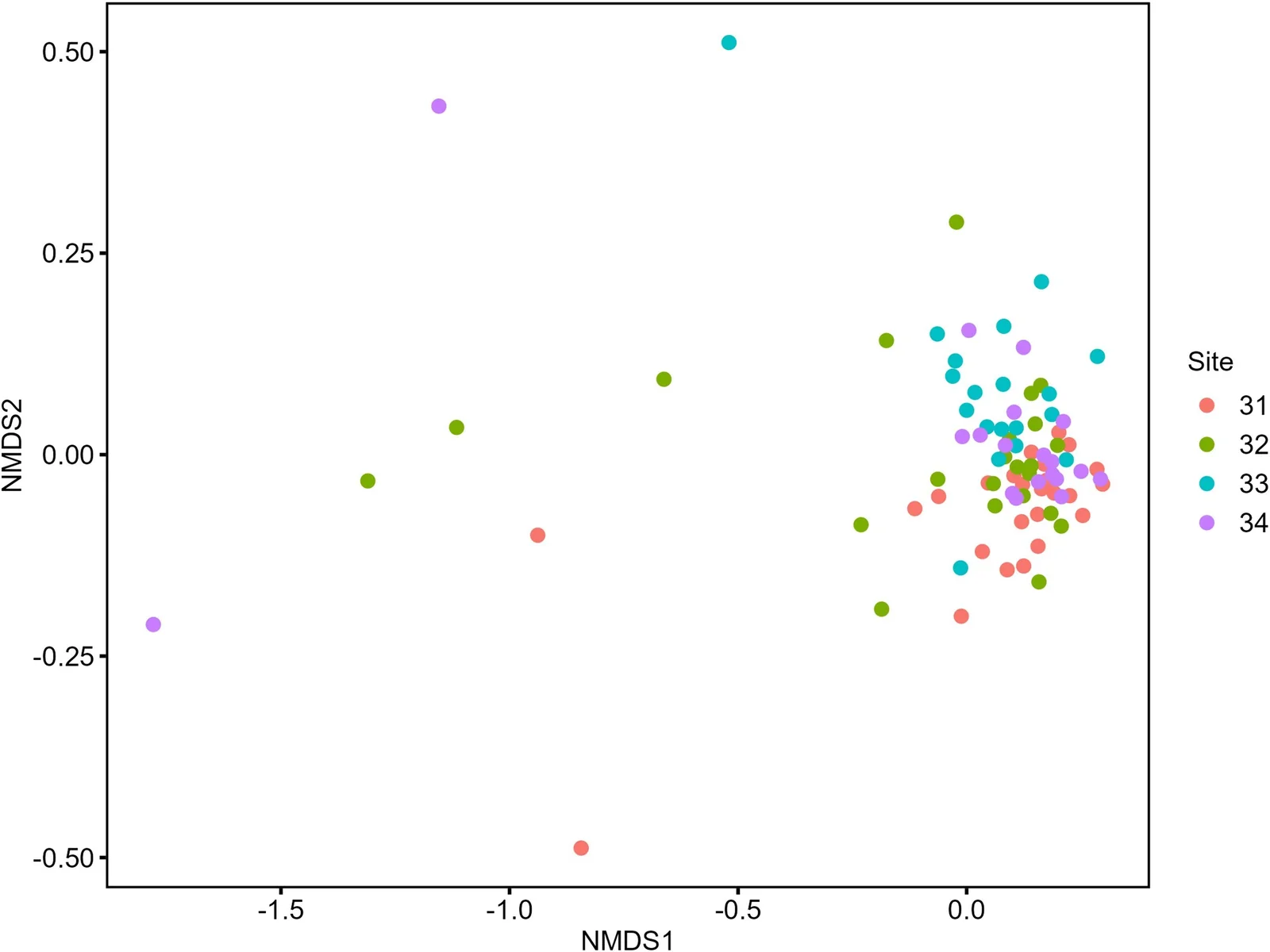
Non-metric multidimensional scaling plot showing relatedness of individual sample depths when rarefied to an even depth with a 1000 minimum read filter (stress = 0.116). Each datapoint represents a depth within a core.

Significant differences were observed between cores with no applied minimum read filter, and with filters of 1000 and 2000 minimum reads (*P* = 0.0001). The location of sampling had a significant impact on the identified microbial community. 7.4%–9.3% of variation in microbial community composition correlated with the location of sampling (Table 2). Beta dispersal tests were non-significant, indicating that the assumptions of the PERMANOVA were met, increasing confidence that these results reflect true compositional variation rather than being an artefact of unequal dispersion. In addition, pairwise comparisons showed that all cores were significantly different from each other, when analyzed with a minimum read count of 1000, post-rarefication (Table 3). However, the NMDS ordination did not reveal clearly distinct clusters based on sampling location (stress = 0.116, Figure 5).

**Table 2:** Observed R^2^ and *p*-values of the influence site, depth and site plus depth have on the identified community profile.

	R^2^	*P*-value
Site (no minimum read filter)	0.074	0.0001
Depth (no minimum read filter)	0.02	0.0146
Site × Depth (no minimum read filter)	0.055	0.0004
Site (minimum read filter of 1000)	0.096	0.0001
Depth (minimum read filter of 1000)	0.034	0.0006
Site × Depth (minimum read filter of 1000)	0.068	0.0001
Site (minimum read filter of 2000)	0.093	0.0001
Depth (minimum read filter of 2000)	0.032	0.001
Site × Depth (minimum read filter of 2000)	0.071	0.0001

**Table 3:** PERMANOVA results with a minimum read number of 1000, post-rarefication, showing significant differences in community profile composition between all cores.

		*P*-value	Adjusted *P*-value
31	32	0.0032	0.0064
31	33	0.0001	0.0003
31	34	0.0185	0.0185
32	33	0.006	0.009
32	34	0.011	0.0132
33	34	0.0001	0.0003

### Depth in core comparison

Within individual cores, there was a significant difference in the microbial community composition associated to depth (Table 2). This trend was observed at all minimum read thresholds. Across all cores, there was no significant difference in microbial community composition between volcanic and non-volcanic horizons (R^2^ = 0.009, *P* = 0.834). There were, however, outliers within specific cores at specific volcanic horizon-associated depths, despite those associated depths not varying from the whole core. Table 4 shows a list of volcanic horizons, associated depth per core (if available) and outlying orders in comparison to the whole core.

**Table 4:** All known volcanic horizons, adapted from Anschütz et al. (2011), with the approximate deposition layer depth for each core and any of the 20 most abundant orders that were outliers in comparison to the wider core. The detection of Enterobacteriales should be interpreted cautiously, as members of this order have been reported in both studies of snow and ice depositional layers and as a source of laboratory-associated contamination (Zhong et al. 2018).

Volcanic horizon	Core	Depth (m)	Outliers
Agung (1963)	Core 31	3.00	Clostridiales
	Core 32	2.37	Enterobacteriales, Rhodobacterales
	Core 34	3.22	Clostridiales, Flavobacteriales, Oceanospirillales
Krakatau (1883)	Core 31	7.62	Caulobacterales, Oceanospirillales, Burkholderiales, Bacillales, Actinomycetales, Clostridiales
	Core 32	6.93	Rhodobacterales, Burkholderiales, Flavobacteriales, Xanthomonadales
	Core 33	5.63	Enterobacteriales
	Core 34	9.22	—
Tambora (1815)	Core 31	10.98	Enterobacteriales
	Core 32	10.33	—
	Core 33	8.98	Xanthomonadales, Bacteroidales, Sphingobacteriales
	Core 34	13.57	—
Unknown (1695)	Core 31	16.98	Rhizobiales, Bacillales, Actinomycetales
	Core 32	16.03	Enterobacteriales
	Core 33	13.76	—
	Core 34	—	—
Deception Island (1641)	Core 31	20.34	Flavobacteriales, Xanthomonadales, Caulobacterales
	Core 32	16.92	Rhizobiales, Bacillales, Actinomycetales
	Core 33	17.03	Rhizobiales
	Core 34	—	—
Unknown (1622)	Core 31	22.49	Rhodocyclales
	Core 32	20.39	Rhizobiales
Huaynaputina (1600)	Core 31	25.33	Enterobacteriales, Xanthomonadales

### Differences in alpha diversity

At all minimum read count filters for both raw and rarefied data Shannon index was significantly higher in Core 31 than in Core 33, suggesting that this observation is robust to uneven sequencing depth and likely reflects true biological variation rather than technical variation. Core 31 also had significantly higher Shannon diversity than Core 32 (apart from when no minimum read count filter was applied and the data was subsequently rarefied to an even depth based on the lowest read count of 157 reads). This suggested that very low sequencing reads reduce the statistical power to detect differences, and that at least 1000 reads per sample is required for detection. In addition, at the higher minimum read counts (5000 and 7500) there was a significant difference in Shannon index between Core 33 and Core 34. However, as this difference is highly process-dependent it should be interpreted cautiously, highlighting the importance of data quality and handling in the outcome, which can be challenging in low biomass environments.

Simpson’s index was significantly higher in Core 31 than in Core 33 for all minimum read count thresholds. With no minimum read depth filter and rarefied data Simpson’s index was marginally higher in Core 31 than in Core 33 (*P* = 0.054), becoming significant when a minimum read count of 1000 reads or more was applied. Significant differences in Simpson’s index between Core 31 and Core 32 were highly filter-dependent so should be interpreted cautiously.

### Core geochemistry

**Figure 6:**
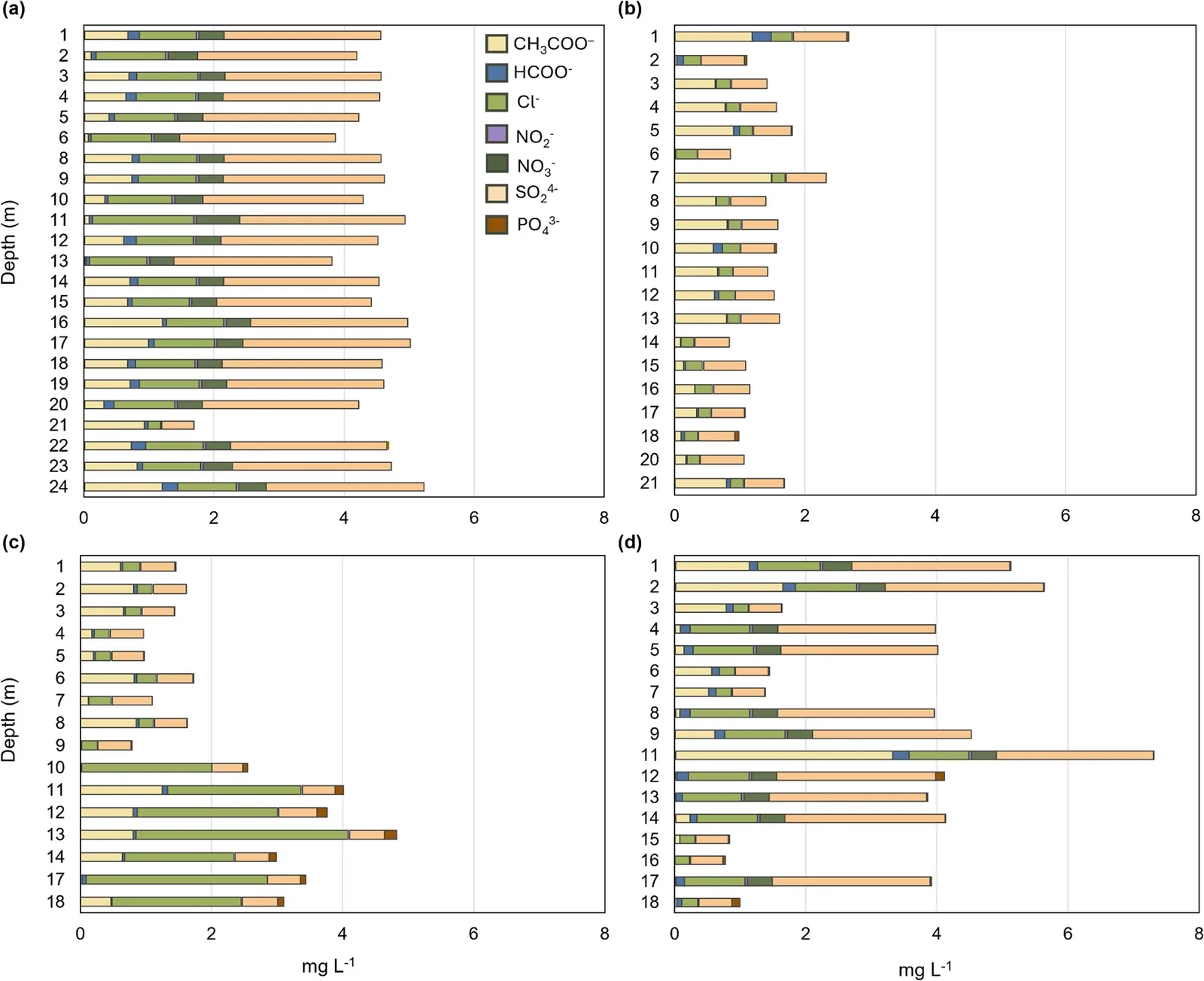
Chemical profile of major anionic and organic compounds detected at different depths in (a) Core 31, (b) Core 32, (c) Core 33, and (d) Core 34. Fluoride, formate, nitrite, nitrate, sulphate, phosphate showed significant correlation to observed differences in the microbiome.

The total combined ion concentrations were 1–8 mg l^−1^ (Figure 6), similar to previous studies that suggested ion concentrations are generally less than 1 mg l^−1^ across the EAIS (Lorius et al. 1969); however, Core 31 and Core 34 had the highest total combined concentrations compared to Core 32 and Core 33 (Figure 6a and d). All investigated ions, except acetate, showed significant differences between cores (Table 5). Generally, all cores exhibit similar ion concentrations, with Cores 31, 32 and 34 dominated by sulphate (2.3, 0.6 and 1.7 mg l^−1^ on average, respectively), whereas Core 33 is dominated by chloride (1.1 mg l^−1^ on average compared to 0.5 mg l^−1^ of sulphate). Core 33 also exhibited the highest phosphate concentrations of all cores, though still low (average of 0.06 mg l^−1^), whilst negligible amounts were detected in Cores 31, 32, and 34 (0.002, 0.009, and 0.03 mg l^−1^, respectively). Full results of ion chromatography are also available in SIT1.

**Table 5:** Kruskal–Wallis tests showing all investigated ions. All except acetate showed significant differences between cores. Pairwise Wilcoxon rank sum test with continuity correction, adjusting using the Holm method, is available (SIT2).

Ion	*P*-value
Fluoride	< 0.0001
Acetate	0.2083
Formate	< 0.0001
Chloride	< 0.0001
Nitrite	< 0.0001
Nitrate	< 0.0001
Sulphate	< 0.0001
Phosphate	< 0.0001

**Figure 7:**
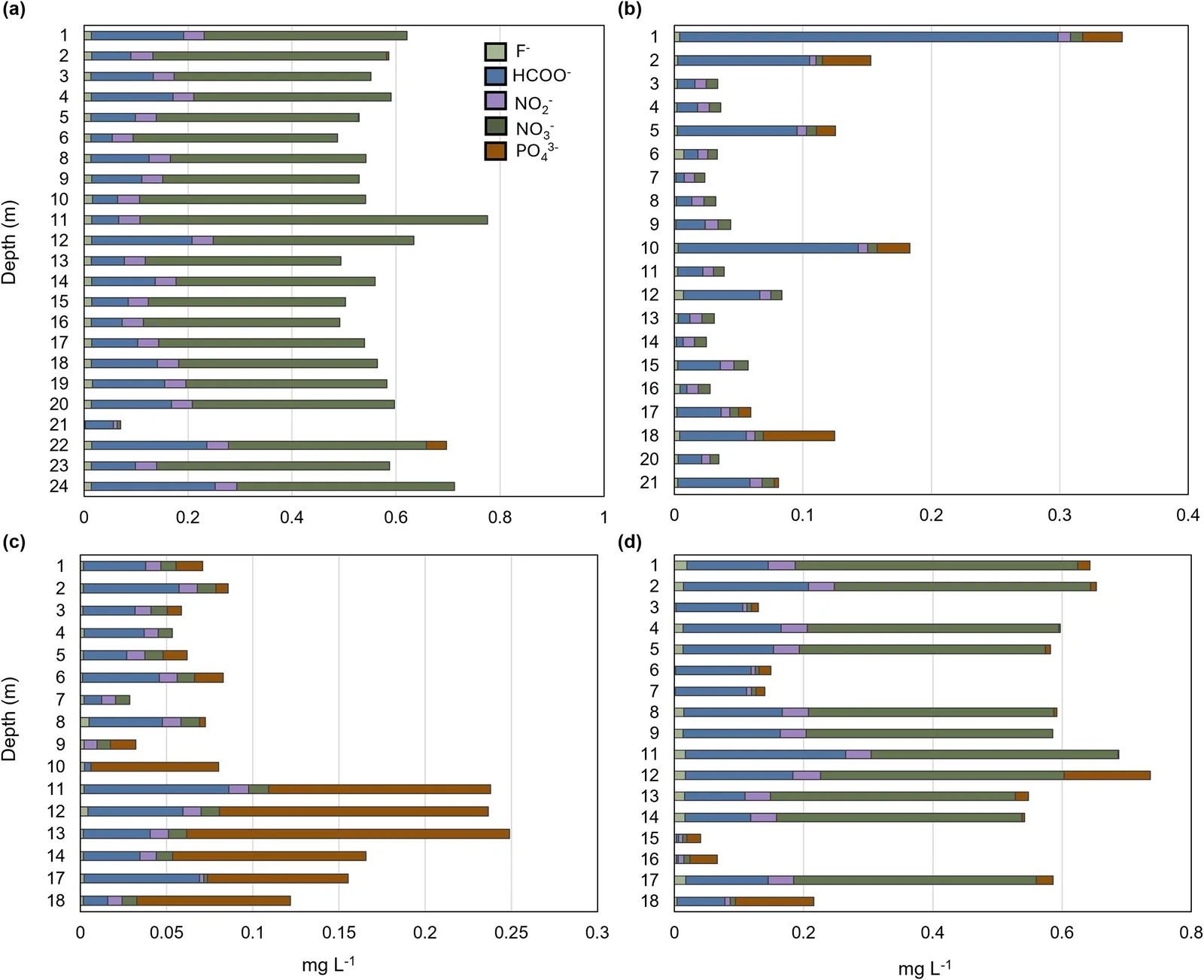
Chemical profile of major anionic and organic compounds exhibiting relatively low concentrations, detected at different depths in (a) Core 31, (b) Core 32, (c) Core 33, and (d) Core 34.

Downcore, total ion concentrations were consistent in Core 31, except with acetate (*P* = 0.0210) and there were no observed differences in Core 32 (Figure 6a and b). Core 33 showed a clear increase in ion concentrations downcore, coinciding with the highest phosphate concentrations of 0.08–0.2 mg l^−1^ occurring in the lower 8 m of the core (*P* = <0.0041), with chloride and nitrate also showing significant changes in concentration with depth (*P* = <0.0011 and *P* = 0.7320), (Table 6 and Figure 6c). Core 34 showed differences in acetate with depth (0.0030) (Figure 6d). Instead, there were sections with markedly higher solute concentrations, particularly at 11 m. This increase coincided with a shift in solute dominance from sulphate to the organic compound acetate. Measured ions that exhibit low concentrations, relative to ions such as sulphate, are shown in Figure 7.

**Table 6:** Observed differences within core samples of each investigated ion (Spearman’s rank correlation). Core 31: moderate positive correlation between acetate and depth (rh0 = 0.482). Core 33: strong positive correlation between chloride and depth (rho = 0.756) and phosphate and depth (0.711), and strong negative correlation between nitrite and depth (rho = −0.747). Core 34: strong negative correlation between acetate and depth (rho = −0.674).

Ion	Core 31	Core 32	Core 33	Core 34
Fluoride	0.3133	0.4322	0.4437	0.8389
Acetate	0.0210	0.0849	0.7049	0.0030
Formate	0.4446	0.5267	0.8525	0.0572
Chloride	0.2365	0.1031	0.0011	0.3880
Nitrite	0.3980	0.9195	0.7320	0.2596
Nitrate	0.9838	0.4367	0.0013	0.0992
Sulphate	0.9334	0.9873	0.3262	0.6873
Phosphate	0.5598	0.7393	0.0041	0.0585

In a rarefied subset of samples with complete ion concentration data and analyzed with a 1000 minimum read count filter, 21% of observed variation in microbial community composition was explained by differences in the ion concentration profiles (PERMANOVA: *P* = 0.0001). The significant pattern was also observed for all other minimum read count filters. Fluoride, formate, nitrite, nitrate, sulphate, and phosphate were significantly correlated with the NMDS ordination (Table 7), with phosphate showing the statistically strongest correlation (R^2^ = 0.17). With no minimum read count only phosphate was significantly correlated with the ordination (R^2^ = 0.19, *P* = 0.004).

**Table 7:** The correlation between each analyzed compound and NMDS ordination (post-rarefication and with a 1000 minimum read filter applied).

Anion or organic	R^2^	*P*-value
Fluoride	0.1255	0.0048
Acetate	0.0268	0.3315
Formate	0.0949	0.0384
Chloride	0.0351	0.2637
Nitrite	0.1050	0.0125
Nitrate	0.1407	0.0032
Sulphate	0.1270	0.0031
Phosphate	0.1656	0.0145

### Identification of dominant taxa

With the parameters outlined (≥80% coverage across samples with ≥1% total relative abundance), Proteobacteria, Firmicutes, Actinobacteria, and Bacteroidetes represented the dominant phyla across this study. At the taxonomic rank of order, the taxa that met this threshold were Actinomycetales, Pseudomonadales, Bacillales, Lactobacillales, Burkholderiales, Rhizobiales, Sphingomondales, Clostridiales, Flavobacteriales.

## Discussion

### Community composition differences across cores

Despite relatively uniform accumulation rates and broadly similar glaciological conditions across the high-altitude inland plateau of the EAIS (Malard et al. 2019, Anschütz et al. 2011), microbial community composition varied significantly between cores. These observed differences in community structure were unlikely to be due to uneven sequencing depth, as read numbers did not significantly differ between cores. The location of sampling was significantly associated with the microbial community composition (PERMANOVA, *P* = 0.0001), explaining 9.6% of the observed variation. Although the NMDS ordination value indicated a suitable 2D representation (0.116), distinct clustering of cores was not observed. This suggested that the spatial variation observed may occur gradually, rather than distinct microbial assemblages with little overlap. The relatively low proportion of variation correlated with site indicated that the geographic location alone was not the primary driver of microbial communities across the transect area. Instead, the observed heterogeneity is more likely a combination of local depositional histories, microbial source inputs, environmental filtering and preservation processes. These findings show that microbial communities within firn of the high-altitude inland EAIS plateau are spatially heterogeneous.

### Downcore (depth-associated) changes in biodiversity

The microbial assemblage within individual cores varied significantly, and there were also significant differences between specific depths of different cores (site × depth) (Table 3). This statistically significant pattern suggests that site-specific depositional, environmental, and preservation processes may influence vertical shifts in community composition. Importantly, these identified differences were not driven by variation in sequencing depth or ASV richness, with the patterns of diversity remaining consistent across taxonomic ranks and minimum read thresholds. These depth-associated changes extend beyond the surface layers of Antarctic snow and ice, providing substantially longer temporal and depositional records.

As depth of sample broadly correlates with age and depositional history (Anschütz et al. 2011), downcore changes in microbial community likely reflect variation in atmospheric inputs, accumulation history and post-depositional processes. To test if discrete depositional events contribute to the overall shifts in downcore microbial population structure, depths that corresponded to known volcanic horizons, as described by Anschütz et al. (2011), were specifically analyzed for changes in relative abundance. The volcanic horizons were Agung (1963), Krakatau (1883), Tambora (1815), an unknown horizon (1695), Deception Island (1641), a second unknown horizon (1622), and Huaynaputina (1600). Several bacterial orders showed deviations in their relative abundance at volcanic horizon-associated depths across one or more cores. These included Enterobacteriales, Rhizobiales, Flavobacteriales, Caulobacterales, and Clostridiales. However, these deviations were not consistently reproduced between cores, as only Clostridiales, Burkholderiales, and Rhizobiales reoccurred at equivalent volcanic horizons across multiple cores. These deviations were also not indicative of volcanic deposition producing consistent or predictable shifts in the firn microbiome, as there were no significant differences detected in overall community composition between volcanic and non-volcanic horizon-associated depths (PERMANOVA, 0 = 0.834). Volcanic horizons instead influence the relative abundance of specific individual taxa at specific individual depths and locations, without driving broader community changes.

### Local ion composition correlated with the firn microbiome

The microbial communities identified within this study may also respond to spatial variation in major ion availability (Falconer et al. 2015), including fluoride, formate, nitrate, nitrite, sulphate, and phosphate. In low biomass environments microorganisms are sensitive to geochemical gradients, with even small differences in ion concentrations influencing community structure by limiting or promoting the growth of microbial populations (Garcia-Lopez et al. 2022, Varliero et al. 2023). Such sensitivity to geochemical heterogeneity may explain observed correlations between the microbiome and individual ions detected here (Falconer et al. 2015).

Depth associated variation in ion concentration was observed within cores. Acetate concentration in Core 31 showed significant variation, while chloride, nitrate, phosphate all varied with depth in Core 33 (Table 6). Notably, acetate and chloride showed no significant correlations with the NMDS ordination. Whereas phosphate concentrations, despite being the lowest measured ion in terms of absolute concentration (Figure 7), showed the strongest correlation with the ordination, potentially indicating that it may be an important geochemical correlate of microbial community structure. Phosphate is a source of phosphorus, a biologically relevant compound required for phospholipid biosynthesis, nucleic acids and cellular metabolism. In low-biomass environments, such as Antarctic firn, relatively small levels of variation in the availability of essential compounds, such as phosphate, may influence the persistence or relative abundance of microbial populations (McCutcheon et al. 2021, Schmidt et al. 2022). Observed variation in phosphate availability may influence the relative competitiveness, persistence or activity of components of the microbial community, potentially contributing to the observed differences between and within cores. This is consistent with previous studies demonstrating that phosphate can be a limiting nutrient for microbial growth in Antarctic glacial ice and snow, even at low absolute concentrations (Stibal et al. 2009), although the observed association may also reflect covariance with other environmental or depositional factors. Studies of firn and shallow ice cores in other glacial environments have similarly reported relationships between inorganic ion profiles and microbial community composition, particularly phosphate, suggesting that geochemical variability preserved in the firn column reflects, at least in part, biological as well as depositional signals (Stibal et al. 2009, Antony et al. 2012, McCutcheon et al. 2021, Henley et al. 2023, Keuschnig et al. 2023). The particularly strong correlation of phosphate here therefore is biologically plausible and may indicate that variation in phosphorus availability contributes to differences in the microbial community structure.

In the subset of samples with available ion concentration data, local ion concentration profiles correlated with 21% of observed microbial population variation, exceeding the proportion correlated with location of sampling alone (R^2^ = 0.103). This indicated that spatial differences in ion concentrations were associated with microbiome variations across these cores. Such differences in ion concentrations may arise through sediment entrainment and higher nutrient loads associated with variable atmospheric inputs and katabatic wind systems to the EAIS surface (Paun et al. 2019, Toubes-Rodrigo et al. 2021). Aeolian deposition has been shown to establish and differentiate microbial communities in snow and glacier ice, creating depth-dependent variation in community structure (Xiang et al. 2009, Chen et al. 2022). Post-depositional redistribution and loss of volatile ions can also occur, including nitrate and chloride, which may contribute to the spatial heterogeneity in ion concentrations observed here (Noro and Takenaka 2020, Chen et al. 2022). These associations highlight the potential importance of geochemical variability at very fine spatial and concentration scales in structuring microbial community patterns, and suggest that shifts in atmospheric deposition or meteorological systems could alter the surface geochemistry and hence influence microbial community composition. The observed relationships remain correlative, and causal mechanisms cannot be inferred from the current dataset alone.

### Dominant taxa identification

The dominant taxa identified in this study consisted of the phyla Proteobacteria, Firmicutes, Actinobacteria, and Bacteroidetes, with Actinomycetales, Pseudomonadales, Bacillales, Lactobacillales, Burkholderiales, Rhizobiales, Sphingomondales, Clostridiales, Flavobacteriales representing the dominant microorganisms at the order level. Several of these taxa showed uneven distributions among cores and with depth. Rhizobiales, in particular, contributed strongly to deeper sections of Cores 32 and 33, representing one of the clearest compositional shifts between samples (Figure 3b). This, however, was also uneven, with the shift in Core 33 occurring ∼12 m, but occurring at ∼16 m in Core 32. Such differences may reflect local depositional histories, source contributions, preservation processes or even volcanic horizons.

The identification of these taxa as members of a dominant microbiome reflects phyla commonly associated with adaption to cold and oligotrophic conditions with evidence of atmospheric dispersion (Bottos et al. 2014). Proteobacteria was the only phylum consistently identified across both this and other similar shallow ice studies of the Antarctic (Malard et al. 2019, Stoppiello et al. 2023, Nowak et al. 2024, Parro et al. 2025). The repeated occurrence of Proteobacteria across geographically distinct samples suggests that members of this phylum may be widespread components of the EAIS cryosphere, although it may also reflect the broad diversity within and size of the lineage (Kersters et al. 2006). Across these studies, only five phyla were identified as components of a dominant microbiome: Proteobacteria, Firmicutes, Actinobacteria, and Bacteroidetes and Cyanobacteria (Malard et al. 2019, Stoppiello et al. 2023, Nowak et al. 2024, Parro et al. 2025) (Table 8). Differences in the dominant taxa identified between Dome Fuji and Dome C-associated studies might reflect regional differences in atmospheric transport pathways, air mass origin and local depositional histories. These taxa may therefore represent a core cryosphere-associated microbiome across surface snow, shallow ice and firn environments of the Eastern Antarctic.

**Table 8:** The identified dominant microbiomes of shallow ice studies from both Western and Eastern Antarctica. Ticks indicate identification as part of a dominant microbiome, with a cross demonstrating it was not. The rank of Phyla was selected as this was commonly reported across studies in relation to dominant taxa.

Phylum	This study	Parro et al. (2025)	Nowak et al. (2024)	Stoppiello (2023)	Malard et al. (2019)
Proteobacteria	✓	✓	✓	✓	✓
Firmicutes	✓	✓	x	✓	x
Actinobacteria	✓	✓	✓	x	x
Bacteroidetes	✓	✓	x	✓	x
Cyanobacteria	x	x	✓	✓	x

### Patterns in Eastern Antarctic high-altitude inland plateau firn

The microbial community structure identified across this study showed significant variation across cores, with depth in cores, and between specific depths in different cores. The results of this study demonstrated spatial heterogeneity of the microbial community profile across the EAIS. Observations of localized microbial diversity were consistent with previously reported findings (Malard et al. 2019, Parro et al. 2025), supporting the hypothesis that microbial populations across the EAIS are influenced by local environmental conditions and depositional histories. Despite broadly similar accumulation rates (Anschütz et al. 2011), the significant interactions between site and depth suggest that the deposition of microbes across the EAIS is not uniform, and may reflect local depositional histories. Significant associations were also observed between the microbial communities identified and measured ion concentrations. Although these correlations were significant, they are not direct evidence of biological responses, and may arise from shared environmental factors.

The spatial diversity of microbial communities observed in this study is consistent with the influence of large-scale atmospheric systems and local depositional histories. Although the interior of the EAIS experiences generally weak near-surface winds, katabatic wind systems, driven by ice-sheet topography transports surface snow and aerosols towards coastal regions (Wang et al. 2023, Cable et al. 2026). This may contribute to gradual shifts in microbial inputs along inland-coastal gradients, consistent with the absence of sharply defined clustering in community composition. However, location of coring alone explained <10% of variation, suggesting that large-scale circulation alone is not the main determinant for the observed patterns. Observed variation between the microbiome in inland EAIS cores may reflect the origin, pathways and mixing between inland and coastal air.

Across the EAIS, the measured ion concentrations are generally low but varied spatially, whereas cryoconite holes in blue-ice areas exhibit relatively higher measured concentrations, correlating with local microbial community compositions and potential biogeochemical cycling (Nowak et al. 2024, Parro et al. 2025). The variability of microbial communities present in Antarctic surface snow and ice environments have been associated with local chemical concentrations in previous studies (Nazaries et al. 2013, Nowak et al. 2024, Parro et al. 2025), but the extent to which these relationships reflect biological responses, rather than shared depositional histories, remains unclear. The measurement of local ion concentrations, however, may provide context for the interpretation of observed microbial distributions within Antarctic firn.

## Conclusions

The microbial community composition of firn across this long-range high-altitude transect of the EAIS exhibited clear spatial structuring, with variation observed both between different cores and with depth within cores. Variation in local ion concentrations showed a stronger association with microbial community compositional differences than either site or depth alone, with phosphate showing the most pronounced correlation among the analyzed ions. At the regional scale, core microbial communities within the Dome Fuji region were broadly similar, despite variability between sample cores. Across comparable studies of Antarctic shallow-ice studies, Proteobacteria was the only phylum to be detected across multiple major sectors of the EAIS, suggesting the presence of a limited, but stable, core component of the microbial community. These findings demonstrate that high-altitude, inland firn of the EAIS host a spatially heterogeneous microbial community, with compositional variation observed both geographically and with depth. Volcanic horizon layers did not influence the composition of microbial populations in predictable or consistent patterns, indicating that discrete depositional events and accumulation history rates were not the primary drivers of the observed variation. Instead, microbial heterogeneity likely reflects a combination of atmospheric transportation, depositional history and fine-scale geochemical variation, highlighting the importance of incorporating local environmental variables in interpreting microbial distributions across Antarctic surface and shallow-ice environments.

## Data Availability

Sequence reads are available in the Sequence Read Archive (SRA) under BioProject: PRJNA1442210.
